# Sinonasal Cancer and Occupational Exposure in a Population-Based Registry

**DOI:** 10.1155/2013/672621

**Published:** 2013-09-04

**Authors:** Carolina Mensi, Dario Consonni, Claudia Sieno, Sara De Matteis, Luciano Riboldi, Pier Alberto Bertazzi

**Affiliations:** ^1^Department of Preventive Medicine, Fondazione IRCCS Ca' Granda—Ospedale Maggiore Policlinico, Via San Barnaba 8, 20122 Milan, Italy; ^2^National Heart & Lung Institute, Department of Occupational & Environmental Medicine, Imperial College London, London SW3 6LR, UK; ^3^Department of Clinical Science and Community Health, Università Degli Studi di Milano, Via San Barnaba 8, 20122 Milan, Italy

## Abstract

We examined occupational exposures among subjects with sinonasal cancer (SNC) recorded in a population-based registry in the Lombardy Region, the most populated and industrialized Italian region. The registry collects complete clinical information and exposure to carcinogens regarding all SNC cases occurring in the population of the region. In the period 2008–2011, we recorded 210 SNC cases (137 men, 73 women). The most frequent occupational exposures were to wood (44 cases, 21.0%) and leather dust (29 cases, 13.8%), especially among men: 39 cases (28.5%) to wood and 23 cases (16.8%) to leather dust. Exposure to other agents was infrequent (<2%). Among 62 subjects with adenocarcinoma, 50% had been exposed to wood dust and 30.7% to leather dust. The proportions were around 10% in subjects with squamous cell carcinoma and about 20% for tumors with another histology. The age-standardized rates (×100,000 person-years) were 0.7 in men and 0.3 in women. Complete collection of cases and their occupational history through a specialized cancer registry is fundamental to accurately monitor SNC occurrence in a population and to uncover exposure to carcinogens in different industrial sectors, even those not considered as posing a high risk of SNC, and also in extraoccupational settings.

## 1. Introduction

Cancer of the nasal cavity and the paranasal sinuses, referred to as “sinonasal cancer” (SNC), is relatively uncommon in the general population, accounting for less than 1% of all neoplasms and less than 4% of those arising in the head and neck region [1–3]. SNC incidence is around 1 × 100,000 person-years in most developed countries [4]. In the period 1998–2002, the annual incidence rates in the United States were 0.8 and 0.6 × 100,000 in males and females, respectively [5]. In the same period, the RARECARE project, based on about 2200 cases of epithelial tumors of nasal cavities in Europe, showed an age-standardized rate of 0.36 × 100,000 [6]. The incidence rates in 1998–2002 estimated by the Italian network of cancer registries (Associazione Italiana Registri Tumori, AIRTUM, http://www.registri-tumori.it/), which covers 40% of the whole population, were between 0.4 and 2.0 in males and 0.1 and 0.5 × 100,000 in females, with about 300 cases expected per year in the whole country; a high variability across Italian regions was reported.

Frequency, anatomical site, and histological type of SNCs vary across geographical areas due to several factors [3]. A history of chronic sinusitis, nasal polyps [7], use of nasal drug preparations, and smoking and occupational history of wood and leather working and nickel refining are reported as risk factors for the development of these tumors. A strong relationship between SNC and exposure to wood, leather dust, and nickel compounds has been established long time ago [8–11] and recently confirmed [12]. Other confirmed or suspected causative factors include hexavalent chromium compounds, welding fumes, arsenic, mineral oils, organic solvents, and textile dust [11, 13–16].

SNCs, particularly adenocarcinomas, are characterized by a high occupational etiologic fraction [17]. For this reason, in Italy, since 2008, their incidence and etiology are compulsorily surveyed in the whole Italian population (about 60 million people) through a nationwide cancer registry (ReNaTuNS), coordinated by the Italian Workers' Compensation Authority (Istituto Nazionale per l'Assicurazione contro gli Infortuni sul Lavoro, INAIL). ReNaTuNS collects information from a network of regional registries to monitor SNC incidence and to establish sources of occupational exposure (http://www.inail.it/). In the Lombardy region, northwest Italy, the most populated (almost 10 million residents at the 2011 census) and industrialized Italian region, the registry was established at the end of 2007. In this paper we present clinical characteristics and sources of occupational exposure for SNC cases recorded in the period 2008–2011. We also calculated Lombardy incidence rates of SNC for the period 2008-2009, in which registration of clinical and exposure information was complete.

## 2. Materials and Methods

### 2.1. Identification and Definition of SNC Cases

The Lombardy registry of sinonasal cancers (LRSNC) collects all SNC cases occurring among subjects with residence in the region (from about 9.6 million people in 2008 to about 9.9 million in 2011) at the time of first diagnosis.

The primary sources of information on SNC cases are the departments of diagnosis and treatment of SNC in regional hospitals (more than 100), particularly pathology, otolaryngology, maxillofacial surgery, and radiotherapy. Completeness of reporting is checked by periodic linkage made at LRSNC with databases of pathology departments (six-monthly), hospital discharge records (annual), mortality registries (annual), and occupational disease compensation records from INAIL (annual). This complex system virtually ensures complete ascertainment of SNC cases occurring in Lombardy residents and also allows the identification of the few patients admitted in hospitals outside the Lombardy region.

Diagnosis of SNC is established by a panel of experts at LRSNC based on complete clinical information (copy of clinical records, computed tomography and/or magnetic resonance imaging scans, pathology reports) according to the ReNaTuNS guidelines [18]. SNC cases include all newly diagnosed primary malignant epithelial cancers of the nasal cavity, code C30.0 of the International Classification of Diseases, Tenth Revision (ICD-10) and paranasal sinus, ICD-10 codes from C31.0 to C31.9. Histological types are defined according to the World Health Organization (WHO) classification [1].

### 2.2. Exposure Assessment

A standardized questionnaire is administered to patients or their next of kin by trained interviewers within the hospital in which diagnosis was made or at the occupational health services of the local health units in Lombardy region. The questionnaire is designed to obtain a lifetime occupational history, including industrial sectors, plants, jobs, and specific task performed. Coding of the occupational history is performed according to the Italian Classification of Economic Activities. The questionnaire is then reviewed by occupational physicians and industrial hygienists who assess occupational exposure to agents with an established or suspected association with SNC: wood and leather dust, nickel and chromium compounds, polycyclic aromatic hydrocarbons (PAH), and cork dust. Also exposure to these agents in domestic setting or during hobby activities is evaluated. In case of exposure either in occupational or in other settings, the subjects is classified as occupationally exposed.

### 2.3. Statistical Analysis

For the period 2008–2011, to compare the distribution of demographics, clinical characteristics, and occupational exposures among SNC cases across gender and tumor morphology, we used the chi-square test. Age distribution across gender was evaluated using Mann-Whitney *U* test.

For the period 2008-2009, in which case registration was complete, we calculated age-standardized rates of SNC (×100,000 person-years) separately for men and women using as standards either the Italian population in the year 2001 or the European population. Confidence intervals (CI) of standardized rates were calculated according to the formula proposed by Tiwari et al. [19, 20]. Analyses were performed with Stata 12 [21].

## 3. Results

In the period 2008–2011, we recorded 210 SNC cases, 137 (65.2%) among males and 73 (34.8%) among females (Table 1). Median age was around 68 years in either gender. About one-third of the tumors originated from the nasal cavity, one-fourth from the maxillary sinus, and one-fifth from multiple sites. The ethmoid sinus was affected in almost 25% of men and only about 7% of women. More than 40% of tumors were squamous cell carcinomas. Intestinal-type adenocarcinomas were especially frequent in men (26.3%). An interview was obtained for 200 cases (95.2%). The questionnaire was administered directly to the patient in 146 cases (69.5%) and to his/her relatives in 56 (26.6%). Ever smokers were almost 70% among males and about 40% among women. Smokers were more frequent in subjects with squamous cell carcinoma (63.9%) or adenocarcinoma (68.3%) than in subjects with other morphologies (56.1%). Current smokers were more frequent among subjects with squamous cell carcinomas (34.9%) than in subjects with adenocarcinoma (16.7%) or with other morphologies (15.8%). A history of nasal polyps was reported by 18 subjects (8.6%), 15 men and 3 women.

**Figure 1:**
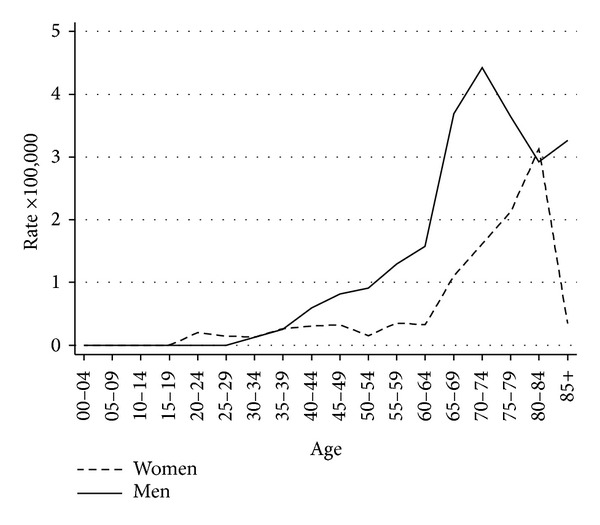
Age-specific sinonasal cancer rates (×100,000 person-years) by gender; Lombardy sinonasal cancer registry, 2008-2009.

In the years 2008-2009, we recorded 82 SNC cases among men and 45 among women. In these years, registration was completed, while for the following two years (55 cases in men and 28 in women), completeness checks are still in progress. Using the Italian 2001 population as standard, in the years 2008-2009, the age-standardized rates (×100,000 person-years) of SNC were 0.9 (95% CI: 0.7–1.1) in men and 0.4 (95% CI: 0.3–0.5) in women. Using the European population as standard, the SNC incidence rates were 0.7 (95% CI: 0.5–0.8) in men and 0.3 (95% CI: 0.2–0.4) in women. Age-specific rates had a peak over 60 years of age in both genders; however, rates began to increase at lower ages, especially in men (Figure 1).

Among men, occupational exposure to any of the six carcinogenic agents mentioned above was identified in 66 subjects (48.2%), while among women, the proportion was 15.1% (Table 2). Wood and leather dust particles were the most prevalent exposures, especially among males (28.5% and 16.8% of subjects, resp.). In women, the corresponding proportions were 6.9% and 8.2%. Exposure to other agents was infrequent (<2%). Seven subjects (six men) had been ever exposed to wood dust and another agent: leather dust (four), nickel compounds (two), and chromium compounds (one subject). We also recorded extraoccupational exposure to a carcinogen for 8 subjects. In the first case, the subject had been exposed to leather dust in the domestic setting, where the mother produced leather buttons. The other seven cases had been exposed to wood dust during hobby activities (construction of furniture and wooden objects or hobby modeling), without using personal protective equipment.

Among subjects with adenocarcinoma, occupational exposure was recorded for the majority (77.4%) of subjects (Table 3). Half had been ever exposed to wood dust and almost one-third to leather dust. One case was exposed to cork dust. Among subjects with squamous cell carcinoma and with tumors of other morphologies, the proportions of subjects ever exposed to any of the six carcinogens were 17.0% and 23.3%, respectively. Exposure to wood or leather dust was about 10% in the case of squamous cell carcinoma and about 20% for other morphologies.

Wood dust exposure occurred mainly (about 80%) in the wood and furniture industry, followed by building construction sectors (8%). The rest of the subjects were exposed in the metal-mechanic sector (construction of wood patterns, use of sawdust in metal polishing) or in a turkey farm (use of sawdust as bedding). Leather dust exposure occurred mainly in boot and shoe manufacture and repair (90%). A few workers were exposed in leather suit or sofa production. Subjects that worked in sofa production were also exposed to wood. Exposure to nickel compounds was recorded in the electroplating industry and in the paint manufacture. Exposure to hexavalent chromium was found for car painters using chromium paints and in the printing industry (use of chromic acid).

The mean length of occupational exposure was 26.6 years (range: 1 to 63) in the 66 men and 15.3 (1 to 38) in the 11 women. The mean latency was 53.3 years (min 18.2, max 74.4) in men and 42.1 years (min 13.6, max 74.6) in women.

Occupational exposure to any of the six carcinogens was relatively more frequent (51.3%) among former smokers (Table 4). Similar patterns were observed for each single agent. Occupational exposure was similarly distributed among the 18 subjects with a history of nasal polyps (8 cases, 44.4%) and among the 192 without (69 cases, 36.9%) (*P* value = 0.47). 

## 4. Discussion

This study describes the findings of LRSNC, a dedicated SNC regional registry. For the first time, incidence of SNCs and the role of exposure to recognized carcinogens were examined in an Italian large population (nearly 10 million people), in a region where shoe/boot factories and repair shops, furniture production, and smelting and metal-mechanic industries are still widely present.

Incidence rates in men were more than twice as high as in women, probably reflecting differences in previous exposure to carcinogens. In this population-wide case series, we found that tumors originate more often (one-third) in the nasal cavities, while localization in maxillary (one-fourth) and ethmoid sinuses (almost one-fifth) was less frequent. This is in agreement with a recent review [2] which considered US and Italian cancer registries data (1998–2002), with a report from The Netherlands registry covering the period 1973–2009 [22], and with UK estimates for 2012 [23]. However, in about 20% of cases, we were not able to determine the site of tumor's origin because multiple sites were involved at first diagnosis. The most frequent morphology in our cases was squamous cell carcinoma. Again, this is in agreement with the mentioned US, Italian, Dutch, and British studies [2, 22, 23]. In our study, we found a closer association between smoking and squamous cell carcinomas and male gender. These were expected findings, in agreement with a European pooled study [24].

The literature estimates of the percentage of SNCs attributable to occupational exposures range from 25% to 41% [25]. In our study, the attributable fraction was 38.5%. In men the proportion of subjects ever exposed occupationally was almost 50%, but also in women it was not negligible (15%). This finding underlines the need to investigate the exposure history, including occupation, of each case of SNC. We found a high frequency of exposure to wood and leather dust, especially among men and among subjects with adenocarcinoma. Although lower, the proportion of subjects with other histology (squamous cell carcinoma or other morphologies) exposed to wood or leather dust was not negligible (about 10–20%). This suggests that not only adenocarcinoma of ethmoid sinus can be induced by wood and leather dust exposure [17, 26].

This study had several merits. First, registration of cases in the LRSNC is quite complete, because the reporting of SNC cases by departments of diagnosis and treatment of SNC is supplemented by an active research which exploits all available information systems (databases of pathology departments, hospital discharge records, mortality registries, and occupational disease compensation records). For this reason, we are confident that the number of cases not identified through these sources is virtually zero. Second, diagnosis of SNC is established by experts based on complete clinical information. Third, diagnostic evaluation is made blind of exposure (the interview is performed after the diagnosis has been verified). Fourth, collection of exposure assessment was successful in 95% of subjects. The major limitation lies in the fact that the years 2010-2011 are still incomplete because some sources of data become available late after case occurrence. Therefore, the exposure profile of the current (in progress) case series might in theory be different from that calculated in the complete case series. However, when we restricted the analysis to the period 2008-2009, the exposure profile was quite similar to that observed in the whole 2008–2011 period (results not shown).

## 5. Conclusion

In conclusion, our study shows that complete collection of cases through a dedicated cancer registry can provide a clear picture of SNC occurrence in a population and of exposure to several relevant carcinogens, in different industrial sectors, including those not considered as posing a high risk of SNC, and also in extra-occupational settings. This allows the recognition of occupational diseases and ultimately compensation of the affected subjects.

## Figures and Tables

**Table 1:** Characteristics of subjects with sinonasal cancer by gender; Lombardy region sinonasal cancer registry, 2008–2011.

	Men		Women		*P* value^a^
	No.	%	No.	%	
No. of subjects	137	100	73	100	
Age (median, min–max)	67.7	31.7–88.5	68.4	21.1–94.9	0.70
Cancer site of origin (ICD-10 code)					
Nasal cavity (C30.0)	44	32.1	27	37.0	0.01
Maxillary sinus (C31.0)	28	20.4	20	27.4	
Ethmoid sinus (C31.1)	32	23.4	5	6.8	
Frontal sinus (C31.2)	0	0.0	1	1.4	
Sphenoid sinus (C31.3)	3	2.2	6	8.2	
Multiple sites	30	21.9	14	19.2	
Cancer morphology					
Squamous cell carcinoma	54	39.4	34	46.6	0.002
Adenocarcinoma, unspecified	7	5.1	3	4.1	
Adenocarcinoma, intestinal type	36	26.3	8	11.0	
Adenocarcinoma, nonintestinal type	6	4.4	2	2.7	
Adenoid cystic carcinoma	8	5.8	12	16.4	
Neuroendocrine carcinoma	10	7.3	0	0.0	
Undifferentiated carcinoma	11	8.0	7	9.6	
Other	5	3.7	3	4.1	
Unknown	0	0.0	4	5.5	
Interview					
Patient	97	70.8	47	64.4	0.06
Relative	37	27.0	19	26.0	
Not performed	3	2.2	7	9.6	
Cigarette smoking					
Never	40	29.2	34	46.6	0.002
Former (>6 mo. before symptoms)	58	42.3	20	27.4	
Current	36	26.3	12	16.4	
Unknown	3	2.2	7	9.6	
Year of diagnosis					
2008 (complete)	32	23.4	23	31.5	0.10
2009 (complete)	50	36.5	22	30.1	
2010 (in progress)	37	27.0	12	16.4	
2011 (in progress)	18	13.1	16	21.9	

ICD-10: International Classification of Diseases, Tenth Edition

^a^From chi-square test, except for age (Mann-Whitney *U* test).

**Table 2:** Occupational exposure to carcinogenic agents among subjects with sinonasal cancer, by gender; Lombardy region sinonasal cancer registry, 2008–2011 (*N* = 200 with interview).

Occupational exposure	Men		Women		*P* value^a^
	No.	%	No.	%	
Never exposed	68	50.7	55	83.3	Reference
Ever exposed (any agent)	66	49.3	11	16.7	<0.001
Wood dust	39	29.1	5	7.6	<0.001
Leather dust	23	17.2	6	9.1	0.02
Nickel compounds	4	3.0	0	0.0	0.08
Chromium compounds	4	3.0	0	0.0	0.08
PAH	2	1.5	0	0.0	0.21
Cork dust	0	0.0	1	1.5	0.27

PAH: polycyclic aromatic hydrocarbons.

^a^From chi-square test (reference: never exposed).

Note: a subject may have been exposed to more than one agent in his/her occupational history.

**Table 3:** Occupational exposure to carcinogenic agents among subjects with sinonasal cancer, by tumor morphology; Lombardy region sinonasal cancer registry, 2008–2011 (*N* = 200 with interview).

Occupational exposure	SCC		Adenocarcinoma		Other		*P* value^a^
	No.	%	No.	%	No.	%	
Never exposed	69	82.1	13	21.3	41	74.5	Reference
Ever exposed (any agent)	15	17.9	48	78.7	14	25.5	<0.001
Wood dust	6	7.1	31	50.8	7	12.7	<0.001
Leather dust	4	4.8	19	31.1	6	10.9	<0.001
Nickel compounds	1	1.2	1	1.6	1	1.8	0.44
Chromium compounds	2	2.4	1	1.6	1	1.8	0.65
PAH	2	2.4	0	0.0	0	0.0	0.46
Cork dust	0	0.0	1	1.6	0	0.0	0.02

PAH: polycyclic aromatic hydrocarbons, SCC: squamous cell carcinoma.

^a^From chi-square test (reference: never exposed).

Note: a subject may have been exposed to more than one agent in his/her occupational history.

**Table 4:** Occupational exposure to carcinogenic agents among subjects with sinonasal cancer, by smoking status^a^; Lombardy region sinonasal cancer registry, 2008–2011 (*N* = 200 with interview).

Occupational exposure	Never smokers		Former smokers		Current smokers		*P* value^b^
	No.	%	No.	%	No.	%	
Never exposed	52	70.3	38	48.7	32	69.6	Reference
Ever exposed (any agent)	22	29.7	40	51.3	14	30.4	0.01
Wood dust	12	16.2	21	26.9	10	21.7	0.10
Leather dust	10	13.5	17	21.8	2	4.3	0.01
Nickel compounds	0	0.0	2	2.6	2	4.3	0.23
Chromium compounds	1	1.4	3	3.8	0	0.0	0.16
PAH	0	0.0	1	1.3	1	2.2	0.47
Cork dust	1	1.4	0	0.0	0	0.0	0.51

PAH: polycyclic aromatic hydrocarbons, SCC: squamous cell carcinoma.

^a^10 subjects with missing smoking history were excluded.

^b^From chi-square test (reference: never exposed).

Note: a subject may have been exposed to more than one agent in his/her occupational history.
